# Fabrication of dendritic PdCu alloy supported on 3D N-doped hollow graphene for efficient ethanol electrooxidation

**DOI:** 10.55730/1300-0527.3530

**Published:** 2022-11-29

**Authors:** Zhijie JIANG, Shuiyuan FU, Wei ZHAO, Xvtang LIU, Fei WANG, Mingyu CUI, Linyang DONG

**Affiliations:** Jiangsu Province Engineering Research Center of Fine Utilization of Carbon Resources, China University of Mining and Technology, Jiangsu, China

**Keywords:** Pd-based binary alloy, ethanol electrooxidation, N-doped hollow graphene, electrocatalysts

## Abstract

Fabricating highly efficient Pd-based nanocatalysts with a well-defined structure is desired for the commercialization of direct ethanol fuel cell (DEFC). Herein, a series of hierarchical three-dimensional N-doped hollow graphene spheres (NHGS) supported dendritic PdCu alloy catalysts Pd_x_Cu_(d)_-NHGS (x: Cu/Pd theoretical molar ratio of 4, 2, and 1) are assembled by one-pot ascorbic acid reduction-immobilization method. Aiming to maximize the Pd utilization and realize the efficient ethanol electrooxidation, this novel electrocatalyst offers potent activity sites and promotes electron and ion kinetics simultaneously. Characterization indicates that the as-obtained Pd_4_Cu_(d)_ alloy nanoparticles with average sizes of approximately 55 nm are evenly dispersed on the NHGS supporting materials obtained by using the SiO_2_ nanospheres template strategy. Three catalysts all exhibit enhanced electrocatalytic activity, of which the Pd_4_Cu_(d)_-NHGS shows the highest mass current activity (2683 mA mg_Pd_^−^^1^), which is 2.59 times of the commercial Pd/C toward ethanol electrooxidation in alkaline medium. Based on the results, we believed that the Pd_4_Cu_(d)_-NHGS could exhibit extensive application prospect in alkaline DEFC.

## 1. Introduction

Direct alcohol fuel cell (DAFC) is a low-temperature proton exchange membrane fuel cell (PEMFC) which could directly use low-carbon alcohols as fuel, such as methanol, ethanol, glycerol, and ethylene glycol [1-4]. Due to the easy storage and high energy conversion efficiency, DAFC has been considered as an ideal power source for portable electronic devices [5]. As a typical renewable fuel, ethanol has attracted extensive attention from researchers because of its high theoretical energy density (8 kWh/kg) [6], extensive biomass-derived availability, low toxicity, environmental friendliness, and low cost [7]. Therefore, direct ethanol fuel cell (DEFC) has developed exponentially in recent decades.

Until now, Pt and Pt-based catalysts have been regarded as the most potent electrocatalysts in electrooxidation of ethanol (EOR) [8, 9]. However, the rarity, prohibitive price, and CO poisoning of Pt all make it arduous to meet the commercial demands [10, 11]. Nowadays, Pd-based electrocatalysts, which are relatively cheaper, and more abundant, emerge as the appropriate alternative to Pt [12, 13], also exhibit excellent electrocatalytical performance and higher affinity for OH- than Pt-based catalysts in alkaline medium [14, 15]. Still, pure Pd catalysts inevitably suffer from low utilization efficiency and inadequate stability because of the poisoning intermediates formed in the reaction [16]. Previous studies have reported that the formation of bimetallic PdM or alloy by doping synergistic secondary metal elements can enhance the electrocatalytic activity and simultaneously reduce the use of precious metals, such as PdAu [17, 18], PdAg [19, 20], PdNi [12, 21], PdSn [22], PdFe [23], PdCu [24–26], etc. The added metal could adsorb oxygen-containing active species at a lower potential to promote the further removal of the intermediate CO-like species from Pd surface. Besides, the synergistic effect that existed in the binary Pd-based alloys with proper atomic ratio could optimize the electronic structure and alter the d-band center of the alloy catalyst compared to the monometallic Pd counterparts, accordingly leading to the improvement of the antipoisoning ability and electrochemical properties [25, 27–29]. Because of the low cost and abundance, the benign metal of Cu, has attracted much attention. Pd nanoparticles possess high surface energy and are very easy to agglomerate, which leads to lower catalyst utilization. Consequently, another alternative approach to fabricating high efficient catalysts is to disperse Pd-based nanoparticles on suitable supports [12, 13].

As a monolayer, hexatomic ring benzene-like structure carbon material, graphene possesses intriguing physical and chemical properties, including high theoretical specific surface area, superior electrical conductivities, excellent structural, and thermal stability. It was a promising carbon-based supporting material for anchoring Pd-based nanoelectrocatalysts in DEFC [30–32]. Two-dimensional (2D) graphene has displayed its unique advantages in current research. However, in practical applications the 2D graphene is unavoidably prone to stacking and self-agglomeration, which reduces the active surface area and hinders the electrolyte from spreading into the inside of the electrode [33, 34]. Therefore, it is feasible to design three-dimensional (3D) graphene with hollow structure through the SiO_2_ hard template method to cope with the above obstacles [35, 36]. 3D graphene possesses larger specific surface area and also could provide more space for the transmission and storage of electrons, ions, and liquids in the electrolyte. Besides, due to its unique hollow structure and intrinsic properties of 2D graphene, it would further improve the conductivity of the graphene network and facilitate the even dispersion of alloy nanoparticles [37–39].

Lee et al. [40] prepared Pd networks supported on the 3D hollow graphene derived from SiO_2_ nanospheres template method and applied it to formic acid electrooxidation. Liu et al. [33] reported the hollow N-doped graphene frameworks (HNGF) for supporting Pd NPs by using poly microspheres (glycidyl methacrylate) as templates. The resulting catalyst demonstrated high electrocatalytic activity and durability for methanol electrooxidation.

Particularly, the incorporation of the N atom into graphene structure, perhaps the most widely chosen method, can further tune the properties of the support since the N atom is of comparable atomic size and possesses available valence electrons to form valence bonds with carbon atoms [11]. Compared to the pristine graphene, doping N atom can improve graphene’s conductivity and help anchor the binary alloy nanoparticles so as to enhance the catalytic activity [41, 42].

Motivated by these latest studies, we found that N-doped hollow structure graphene supported dendritic PdCu alloy catalyst for EOR in alkaline medium was barely reported. Herein, approximately 55 nm dendritic PdCu alloy nanoparticles with various molar ratios were synthesized and loaded on NHGS derived from the template method. Copper was used to lower noble metal Pd usage and to enhance EOR activity through a synergistic effect. The as-prepared PdCu alloy maintained a well-defined dendritic structure after loading on NHGS and other physical morphology, chemical composition, and valence states of the catalysts were analyzed in detail. Additionally, the electrocatalytic performance for EOR of the as-prepared catalysts were investigated by cyclic voltammetry (CV), chronoamperometric (CA), and linear scan voltammetry (LSV) measurements. Electrochemical evaluations demonstrated that Pd_4_Cu_(d)_-NHGS exhibited substantially excellent catalytic activity towards EOR, surpassing those of Pd_2_Cu_(d)_-NHGS, Pd_1_Cu_(d)_-NHGS, and Pd/C. This benefit from the optimized PdCu molar ratios and hollow microsphere structure in NHGS.

## 2. Experimental

### 2.1. Materials

Palladium chloride (PdCl_2_) and tetraethyl orthosilicate (TEOS) was purchased from Macklin Co., Ltd. Copper chloride dihydrate (CuCl_2_.2H_2_O), sulfuric acid (98 wt% H_2_SO_4_), potassium permanganate (KMnO_4_), hydrogen peroxide (30 wt% H_2_O_2_), sodium hydroxide (NaOH), ammonium solution, potassium bromide (KBr), ascorbic acid (AA), ethanol, and urea were purchased from Xi Long Chemical Co., Ltd (Guangzhou, China). Hexadecyltrimethyl ammonium bromide (CTAB, 99%) and 3-aminopropyl-trimethoxysilane (ATPS) were purchased from Aladdin Chemical Reagent Lo., Ltd. 10% Pd/C (Wako Pure Chemical Corporation) was used for comparison. Deionized (DI) water from Millipore was used throughout the experiment. All chemicals were used without any further purification.

### 2.2. Preparation of NHGS

Graphite oxide (GO) and SiO_2_ nanospheres were prepared by the improved Hummers’ method and modified Stöber method [43, 44]. After surface functionalization with ATPS, the functionalized SiO_2_ and GO were added to 50 mL DI water at a mass ratio of 4:1. Then the solution was ultrasonicated for 2 h and constantly stirred for 12 h to let GO fully encapsulate the SiO_2_ via electrostatic interaction. The solution was subsequently centrifuged to remove superfluous GO and then freeze-dried. The product was thermally calcined with urea in a tubular furnace at 700 °C for 2 h under N_2_ atmosphere to obtain NG (nitrogen doped graphene)/SiO_2_ composites. Finally, the composites were heated and refluxed in a 5 M NaOH solution to remove the SiO_2_ template, the resulting product was washed several times with ethanol and DI water. NG was synthesized using the above thermally treated method.

### 2.3. Preparation of PdCu_(d)_ alloy

First, PdCl_2_ and NaCl were dissolved in DI water at a molar ratio of 1:2 and ultrasonicated for 2 h to obtain Na_2_PdCl_4_, then, 0.2 g CTAB, 0.18 g AA, and 0.10 g KBr were dissolved in 15 mL DI water in a 30 mL three-necked flask and stirred for 15 min to blend evenly. The Na_2_PdCl_4_ and CuCl_2_ were added successively with a Pd/Cu molar ratio of 1 (or 2, 4), and the suspension was heated rapidly to 90 °C under stirring for 1 h. After that, the dendritic PdCu_(d)_ alloys were obtained by centrifugation and washed with DI water and ethanol.

### 2.4. Preparation of PdCu_(d)_-NHGS

A series of Pd_x_Cu_(d)_-NHGS were assembled by immobilizing PdCu_(d)_ alloy on the as-prepared NHGS supports. First, 50 mg NHGS was dispersed in 80 mL DI water and sonicated for 20 min, followed by the addition of a certain amount of the above PdCu_(d)_ alloy solution. The mixture was stirred for 4 h at room temperature. The resultant solution was centrifuged and washed three times with acetone and water and dried to obtain Pd_4_Cu_(d)_-NHGS, Pd_2_Cu_(d)_-NHGS, and Pd_1_Cu_(d)_-NHGS, respectively (Pd loadings of 4.1, 4.5, and 4.6 wt% by ICP). For comparison, Pd_4_C_(d)_-NG was obtained with the same procedure (Pd loadings of 4.8 wt% determined by ICP), and Pd-NG was prepared by NaBH_4_ reduction method (Pd loadings of 5.2 wt% by ICP).

### 2.5. Structural characterization

The crystalline structure of catalysts and supports were analyzed by X-ray diffractometer (XRD, Bruker, D8-ADVANCE). The distribution of nanoparticles and microstructure of the as-prepared catalysts were examined by scanning electron microscopy equipped with an energy dispersive spectrometer (SEM-EDS, FEI, Quantan 250) and transmission electron microscope (TEM, FEI, Tecnai G2-F20), and the distribution of the elements were individually scanned. The particle sizes and morphology of template SiO_2_ nanospheres were also observed by SEM. X-ray photoelectron spectroscopy (XPS) was carried out to investigate the surface composition and the chemical valence states of catalysts (Thermo Fisher, ESCALAB 250Xi). The PdCu molar ratio of the alloy samples were determined by laser ablation inductively coupled plasma mass (LA-ICP-MS, Agilent, NWR 213–7900).

### 2.6. Electrochemical measurements

The electrochemical measurements were conducted on CHI 660E workstation in a typical three-electrode cell at room temperature, including Ag/AgCl (saturated KCl) electrode and Pt foil (area: 1 cm×1 cm) electrode served as the reference electrode and counter electrode, and a glassy carbon electrode (GCE: ϕ = 3 mm) as the working electrode. Before the test, N_2_ was blown through the electrolyte for 30 min to degas. The glassy carbon electrode was polished several times with 0.3 and 0.05 μm Al_2_O_3_ powder and washed with ethanol and DI water ultrasonically, and then dried for later use. As for the preparation of the working electrode, a mixture of 3 mg catalyst, 200 μL H_2_O, 75 μL ethanol, and 25 μL Nafion (5 wt%) was ultrasonicated for 15 min to form an ink. Six microliters of the resulting ink was loaded on the GCE and dried at room temperature. The CV, i-t, and LSV tests were conducted in 1 M ethanol+1 M KOH solution at a sweep rate of 50 mV/s.

## 3. Results and discussion

### 3.1. Characterization of catalysts

**Figure 1:**
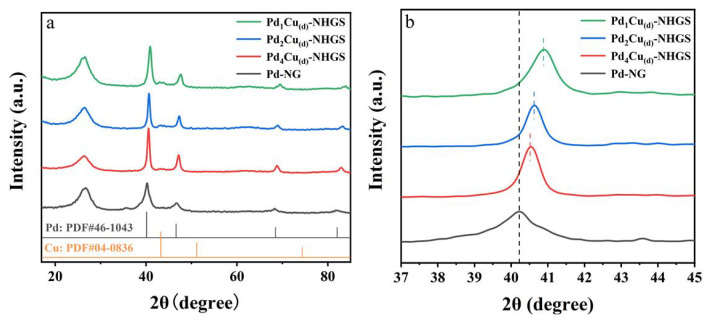
XRD patterns for the synthesized Pd_4_Cu_(d)_-NHGS, Pd_2_Cu_(d)_-NHGS, and Pd_1_Cu_(d)_-NHGS along with Pd-NG (a) and the enlarged patterns at around Pd (111) lattice plane (b).

As shown in Figure 1, The X-ray powder diffraction (XRD) patterns were measured to identify the as-prepared Pd-NG and PdCu_(d)_-NHGS catalysts with different Pd/Cu ratios. The discernible diffraction peak around 26° is attributed to the (002) plane of graphitic carbon, which is the characteristic diffraction peak of graphene materials, consistent with the previous studies [33, 45]. It is found that the peaks of face-centered cubic (fcc) Pd (111) appear prominently around 40.1°, 46.5°, and 68.2° (JCPDS No.46-1043), which are well matched by the diffraction peak of Pd/NG. For PdCu_(d)_-NHGS catalysts (Figure 1a), compared with the standard card of Pd and Cu (JCPDS No.04-0836), all prominent intensive diffraction peaks of catalysts shifted and were located between the standard diffraction peaks of Pd and Cu (Figure 1b), which clearly indicated the alterations of the crystal structure and the generation of alloy [46].

Lattice parameters were obtained by using Bragg’s law for the (111) and (220) planes.


(1) 2d sin θ=n λ

(2) a=2 λ/sin θ

where a is lattice parameter and λ is the wavelength of X-ray. Since the 2θ value of (111) plane may be influenced by the nearby peaks of NHGS, the lattice parameter of alloy has been calculated for all the binary catalysts from the θ values of (220) plane.

Both Pd and Cu possess an fcc crystal structure. According to Vegard’s law, the lattice parameters of the PdCu alloys decreased with the increase of the incorporation of Cu [11, 47]. The lattice parameters of the binary PdCu alloy were calculated based on the XRD data. The mathematical expression can be given as


(3) $${\text{a}}_{\text{al}}={\text{a}}_{\text{A}}\;(1-\text{x})+{\text{a}}_{\text{B}}\;\text{x}$$

Here, a_al_, a_A_, a_B_ and x are the lattice parameters of alloy, host metal A, cometal B, and fractional content of B in the A, respectively. By applying the obtained “a” of PdCu alloy, the degree of alloying (Da) could be calculated.


(4) $$\text{Da}=(\text{a}-{\text{a}}_{0})/({\text{a}}_{\text{al}}-{\text{a}}_{0})$$

where a, a_al_ and a_0_ represent the lattice parameter of the particular alloy catalyst, the theoretically calculated lattice parameter assuming that all the Cu is alloyed, and the lattice parameter Pd NPs, respectively. The calculated d-spacing for the strongest peak (111) of Pd-NG is 2.246 Å, which is consistent with the value in the report (JCPDS-05-0681). The molar ratios of Pd to Cu determined by ICP, the d-spacing, and the lattice parameter of the PdCu alloyed catalysts were summarized and presented in Table S1. The calculated Da of Pd_4_Cu_(d)_-NHGS, Pd_2_Cu_(d)_-NHGS, and Pd_1_Cu_(d)_-NHGS are 54.17%, 59.75% and 71.91%, respectively. With increasing Cu concentrations in Pd lattice, the d-spacing of alloy decreased since Pd possesses a bigger atomic diameter than Cu. The optimal alloying degree indicated the formation of homogeneous solid solution within the Pd and Cu, which can facilitate the modifications of the d band center and electronic properties of alloy [48].

**Figure 2:**
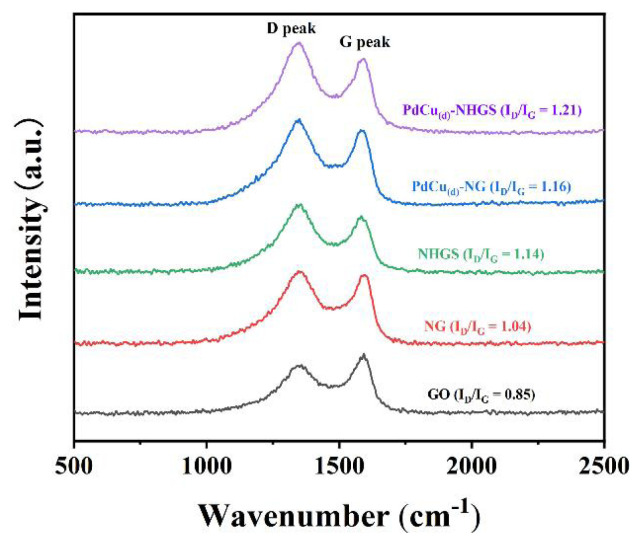
Raman spectra of GO, NG, NHGS, PdCu_(d)_-NG, and PdCu_(d)_-NHGS.

Raman spectroscopy is used to further study the structure of graphene support and catalysts. As shown in Figure 2, the as-prepared GO, NG, NHGS, PdCu_(d)_-NG, and PdCu_(d)_-NHGS all exhibit two characteristic peaks corresponding to the D band and G band of graphene materials around 1344 cm^−1^ and 1590 cm^−1^. The G peak is related to the degree of graphitization, which arises from the in-plane stretching motion of pristine sp^2^ carbon atom pairs. While the D band is associated with the defects which disrupt sp^2^ carbon rings caused by the incorporation of oxygen-containing bonds [11, 25].

Additionally, the intensity ratio of D and G peaks (I_D_/I_G_) can relatively measure the degree of defects in graphite structure. The I_D_/I_G_ values of all the catalysts follow the order: GO (0.85) < NG (1.04) < NHGS (1.14) < PdCu_(d)_-NG (1.16) < PdCu_(d)_-NHGS (1.21). The high I_D_/I_G_ value means an increased defect sites, which will offer more anchoring sites on the support to improve the dispersity and stability of the PdCu alloy, leading to better electrocatalytic performance [11].

**Figure 3:**
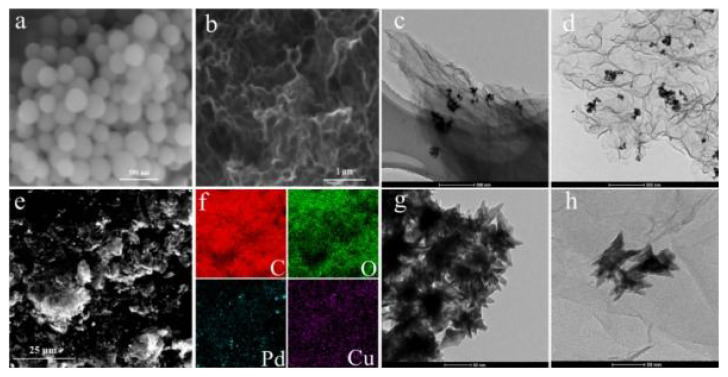
SEM of SiO_2_ and NHGS (a, b), TEM of Pd_4_Cu_(d)_-NG (c), Pd_4_Cu_(d)_-NHGS (d), SEM of Pd_4_Cu_(d)_-NHGS and the elemental mappings of C, O, Pd, Cu (e, f) and PdCu alloy (g, h).

SEM-EDS and TEM were carried out to investigate the morphology and element distribution of the catalysts, supports, and template. Figures 3a and 3b show the SEM images of SiO_2_ and NHGS. The synthesized SiO_2_ nanospheres have a uniform size distribution with an average size of 220–240 nm, this also could be observed in the TEM image of SiO_2_ (Figure S1a). The NHGS support was obtained after the removal of the SiO_2_ template and had a hollow structure. TEM images of Pd_4_Cu_(d)_-NG and Pd_4_Cu_(d)_-NHGS are depicted in Figures 3c and 3d. The NG nanosheets obviously wrinkled and aggregated, which would affect the dispersity of the PdCu alloy. The TEM image of Pd_4_Cu_(d)_-NHGS clearly shows the hollow structure of NHGS. The hollow structure can effectively inhibit the aggregation of graphene nanosheets so as to promote the anchorage and dispersion of alloy nanoparticles.

Furthermore, the SEM-EDS elemental mapping image (Figures 3e and 3f) reveals the even distribution of carbon, oxygen, palladium, and copper. The EDS results indicate that the molar ratio of Pd and Cu in Pd_4_Cu_(d)_-NHGS is approximately 3:1, which roughly agrees with the ICP result. Figure 3h exhibits the well-defined dendritic morphology of the as-prepared PdCu alloy, with the size around 55 nm. And this hierarchical dendritic structure is relevant to the influence of the structure-directing agent, CTAB, which is consistent with the previous studies [46]. Moreover, the PdCu alloy nanoparticles are better dispersed on the NHGS than unsupported PdCu alloy sample (Figures 3g and S1b), suggesting that the highly interconnected hollow structure of graphene is more beneficial for the uniform distribution of the binary alloy nanoparticle and is critical for the facile electron transport simultaneously.

**Figure 4:**
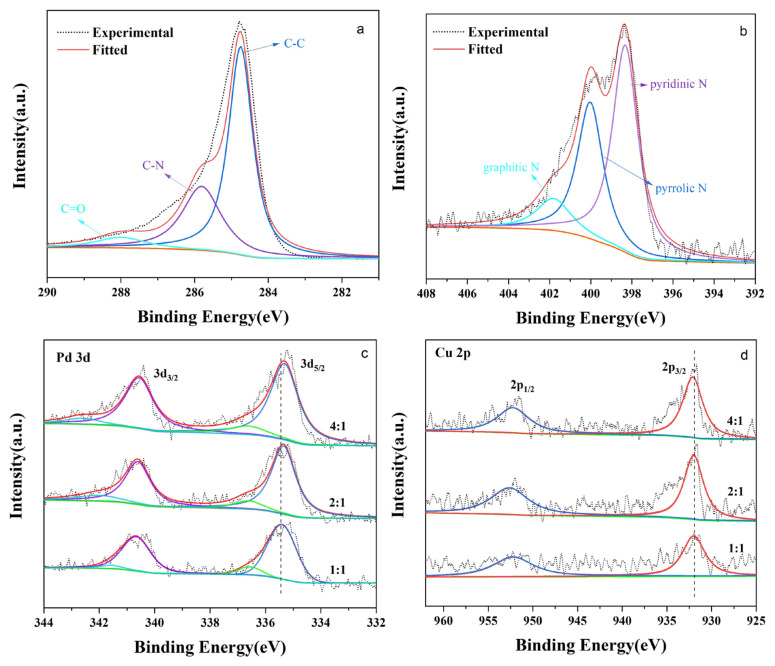
XPS spectra of the C 1s and N 1s (a, b), XPS spectra of Pd 3d and Cu 2p for the PdCu-NHGS (c, d).

To further investigate the structure, XPS characterization was conducted to precisely explore the surface composition and the chemical valence states of the PdCu_(d)_-NHGS catalysts. As shown in Figure 4, it can confirm the existence of C, O, N, Pd, and Cu elements in the PdCu_(d)_-NHGS and Pd-NG composites. The C1s spectra of the supports NHGS consist of three peaks corresponding to C-C, C-N, and C=O (Figure 4a), which are located at binding energy around 284.7 eV, 285.8 eV, and 286.7 eV, respectively [12]. The content of the C=O bond only accounted for a small portion in NHGS, signifying that the oxygen-containing functional groups in GO were mostly reduced during the thermal calcination. The presence of the C–N bond suggests that N atoms were successfully incorporated into graphene structure. The N 1s peak could be deconvoluted into three characteristic peaks at binding energy around 398.4 eV, 399.9 eV, and 401.8eV, corresponding to pyridinic N, pyrrolic N, and graphitic N, respectively [41]. The specific contributions of the above three N species to the total doped nitrogen were calculated based on the fitted N 1s curve (Figure 4b), which were 53.52%, 35.66%, and 10.82%, respectively.

Pd 3d spectra mainly contains two broad peaks emerged at binding energy around 336 and 341 eV in Pd-NG and Pd_4_Cu_(d)_-NHGS as presented in Figure S2. The spectra could be deconvoluted to two groups of peaks at binding energy 335.3 and 336.7, 340.6 and342.2 eV, assigned to the Pd 3d_5/2_ and Pd 3d_3/2_ states of Pd^0^ and Pd^2+^ species on the surface of PdCu_(d)_-NHGS [49]. As shown in Figures 4c and 4d, the deconvolution of Pd and Cu in PdCu_(d)_-NHGS with different Pd/Cu molar ratios were measured, the Pd^0^ and Cu^0^ species are predominant (approximately 70%) compared to the Pd^2+^ and Cu^2+^ in all prepared catalyst. The slight negative shift of the Pd 3d_5/2_ binding energy relative to the Pd-NG is observed, implying the increased electron density. Additionally, the positive shift was observed in Cu 2p_3/2_ binding energy compared to the standard Cu 2p XPS data, which confirms the electron transfer from Cu to Pd [27, 50]. This kind of electron transfer can decrease the d-band energy of Pd, hence facilitating the desorption of the poisonous intermediates and regeneration of the active sites during the electrooxidation of ethanol [51].

### 3.2. Electrochemical analysis

**Figure 5:**
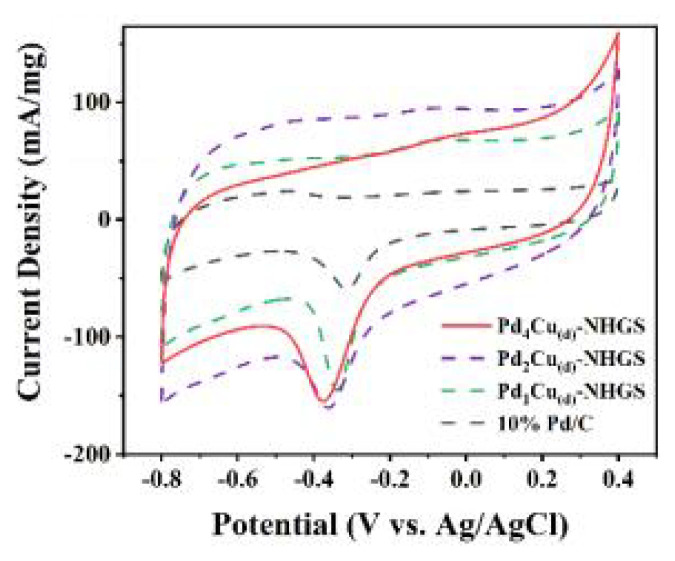
CV curves of different PdCu_(d)_-NHGS and commercial Pd/C in 1 M NaOH solution.

The electrocatalytic performance of the as-prepared catalysts toward EOR in alkaline medium was evaluated by CV tests carried out in 1.0 M NaOH solution with and without 1.0 M C_2_H_5_OH at a scan rate of 50 mV/s. To obtain the electrochemical surface area (ECSA) of the catalysts, the CV curves normalized by the Pd mass were measured in a 1.0 M NaOH solution saturated with N_2_, as shown in Figure 5. The ECSA of catalysts were calculated by the integral area of the reduction of palladium oxide monolayer around −0.25V based on the following equation:


$$\text{ECSA}={\text{Q}}_{0}/405\times \text{m},$$

where m (mg), Q (mC), and 405 (C/cm^2^) represent the mass of Pd on GCE, the total reduction charge of the PdO, and the monolayer PdO reduction charge constant, respectively. The ECSA of Pd_4_Cu_(d)_-NHGS was approximately estimated to be 59.2 m^2^ g^−^^1^, which was larger than those of Pd_2_Cu_(d)_-NHGS (41.5 m^2^ g^−^^1^) and Pd_1_Cu_(d)_-NHGS (37.9 m^2^ g^−^^1^), and 2.4 times higher than commercial 10 % Pd/C (24.2 m^2^ g^−^^1^_Pd_). The higher ECSA of Pd_4_Cu_(d)_-NHGS could be attributed to the uniform distribution of the alloy nanoparticles on NHGS. Moreover, the branched structure of the alloy can also expose more effective active sites, which is also the advantage of the dendritic alloy catalyst.

**Figure 6:**
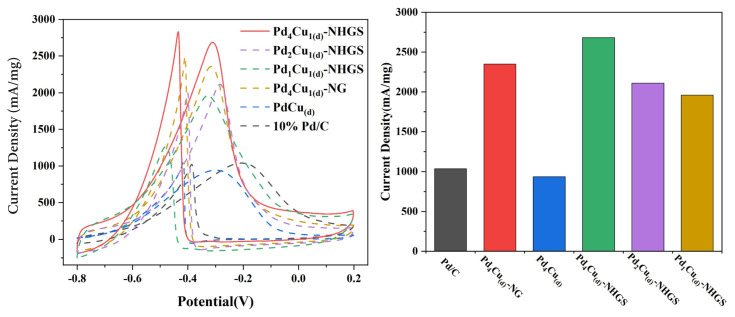
CV curves (a) and the forward peak current density (b) of three PdCu_(d)_-NHGS, PdCu_(d)_-NG, PdCu_(d)_ and commercial Pd/C in 1 M CH_3_CH_2_OH + 1 M NaOH.

The CV curves of the prepared catalyst were measured in N_2_-saturated 1.0 M NaOH + 1M C_2_H_5_OH solution and normalized by the mass activity of Pd, exhibited in Figure 6. Two typical sharp peaks are observed in the forward and backward scans of all catalysts. The characteristic oxidation peak in the forward scan was generated owing to the oxidation of the absorbed ethanol. The peak in the backward scan was ascribed to the oxidation of carbonaceous intermediates, which were incompletely oxidized in the forward scan [12, 52]. The current density in the forward scan of Pd_4_Cu_(d)_-NHGS reaches 2683 mA/mg, which is higher than those of Pd_2_Cu_(d)_-NHGS (2110 mA/mg), Pd_1_Cu_(d)_-NHGS (1958 mA/mg) and Pd_4_Cu_1(d)_-NG (2343 mA/mg) and is 2.86 and 2.59 times of Pd_4_Cu_(d)_ (937 mA/mg) and Pd/C (1036 mA/mg), indicating that the electrocatalytic activity for EOR could be regulated by changing the PdCu alloy composition. And Pd_4_Cu_(d)_-NHGS possesses significantly higher current density at the same Pd loadings than pure PdCu alloy and commercial Pd/C. Also, the current density of Pd_4_Cu_(d)_-NHGS is comparable to or even greater than the recently reported Pd based catalysts, as shown in Table S2.

The presence of Cu in the alloy would facilitate the absorption of oxygenated species, e.g., OH_ads_ at lower potential and thus favors the oxidation of the intermediate product adsorbed on the active sites of the catalyst [32]. Therefore, the synergistic effect of Pd and Cu in PdCu_(d)_-NHGS and the presence of oxygen-containing functional groups in NHGS efficiently reduce the poisoning of intermediate products and release more active sites to promote the reaction. However, high Cu content would lead to excessive OH_ads_ coverage on the surface, hindering the adsorption of ethanol and seemingly rendering the decrease of the catalyst activity [16], which is consistent with the CV results.

**Figure 7:**
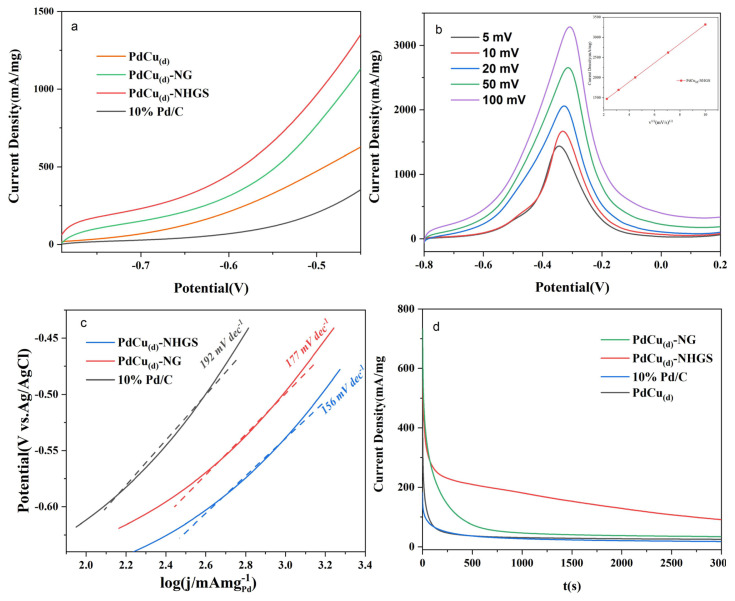
LSV curves of PdCu_(d)_-NHGS, PdCu_(d)_-NG, PdCu_(d)_ and commercial Pd/C in 1 M CH_3_CH_2_OH + 1 M NaOH (a), LSV curves of the Pd_4_Cu_(d)_-NHGS at different scan rates and the inset curve is the linear relationship between peak current density and the square root of the scanning rate (b), Tafel plots of PdCu_(d)_-NHGS, PdCu_(d)_-NG, and commercial Pd/C in 1 M CH_3_CH_2_OH + 1 M NaOH (c), i-t curves of PdCu_(d)_-NHGS, PdCu_(d)_-NG, PdCu_(d)_, and commercial Pd/C in 1 M CH_3_CH_2_OH + 1 M NaOH at −0.45V vs. Ag/AgCl (d).

The onset potential (E_o_) and Tafel slope, which are two essential parameters to evaluate the EOR kinetics, were measured by linear scan voltammetry (LSV) tests. As shown in Figure 7a, compared to commercial 10% Pd/C, all dendritic PdCu-based catalysts show distinct negative-shifted onset potentials, among which the PdCu_(d)_-NHGS exhibited the lowest onset potential, signifying that ethanol was more easily oxidized and the reaction kinetics of alloy was enhanced [53].

As shown in Figure 7b and its inert figure are the LSV curves of the PdCu_(d)_-NHGS at different scan rates and the linear relationship between the square root of scanning rates and the corresponding peak current density. The peak current density of the ethanol oxidation peak increased with the increase of the scan rate, and the corresponding peak potential also shifted positively, indicating that the process is reversible and the kinetics of catalytic EOR in alkaline medium is controlled by the diffusion process [54].

Besides, the kinetics of EOR was further investigated by the Tafel slope, as shown in Figure 7c. The fitted Tafel slopes diminished in the order: commercial Pd/C > PdCu_(d)_-NG > PdCu_(d)_-NHGS. The lower value of Tafel slopes implies the faster transfer of active substances and electrons on the catalyst. Accordingly, the kinetics of OH^−^ adsorbed on the PdCu_(d)_-NHGS is the fastest in the alkaline medium. The hollow structure of NHGS is conducive to the mass transfer process and accelerates the oxidation.

To evaluate the electrochemical stability of these four catalysts toward EOR, the chronoamperometry (CA) tests were measured at −0.45 V vs. Ag/AgCl in a N_2_ saturated 1 M ethanol + 1 M NaOH solution for 3000s. As shown in Figure 7d, the obtained i-t curves reveal that all the polarization currents decay rapidly at the preliminary stage and then deceased gradually [55], and the rates of decay for PdCu_(d)_-NHGS and PdCu_(d)_-NHGS were obviously smaller than the pure PdCu alloy and the commercial Pd/C. At the end of 3000 s, the PdCu_(d)_-NHGS still possessed the highest current density among all these catalysts under the same conditions due to better dispersion on the NHGS supports. Meanwhile, the highest long-term electrocatalytic stability of PdCu_(d)_-NHGS also suggested that the combination of NHGS and alloy enhanced the tolerance against poisonous intermediates for EOR in alkaline media.

## 4. Conclusion

In summary, a series of dendritic PdCu alloy nanoparticles has been synthesized with the help of CTAB as structure-directing agent and ascorbic acid as a reducing agent, and the morphology and dispersion of the catalysts were determined by XRD, XPS, SEM, and TEM. To reduce the consumption of precious metal Pd and simultaneously achieve the synergy of PdCu, the optimum composition of alloy was explored, and the novel graphene material NHGS was introduced as the superior support. According to the electrochemical results, the Pd_4_Cu_(d)_-NHGS exhibited the highest current density among all the as-prepared catalysts. Additionally, it also demonstrated the better kinetics and stability than the Pd-NG and commercial Pd/C toward EOR in alkaline media, which was correlated with the presence of Cu and the N-doped hollow graphene structure. We believe that this work could offer new insights into fabricate high performance Pd-based electrocatalysts for EOR, which will contribute to the development of DEFCs applications.

## Supporting information

Figure S1 TEM images of SiO_2_ nanospheres (a) and Pd_4_Cu_(d)_ alloys (b).

Figure S2 XPS spectra of Pd_4_Cu_(d)_-NHGS and Pd-NG.

**Table S1:** 2θ, d-spacing, lattice parameters, molar ratio, and Da of the catalysts.

Catalysts	Plane	2θ/degree	d-spacing/Å	a/Å	Molar ratio (ICP)	Da/%
**Pd-NG**	111	40.12	2.245	3.884		
	220	68.23	1.379			
**Pd** **_4_** **Cu** **_(d)_** **-NHGS**	111	40.46	2.235	3.845	2.60: 1	54.17
	220	69.02	1.364			
**Pd** **_2_** **Cu** **_(d)_** **-NHGS**	111	40.60	2.228	3.835	2.26: 1	59.75
	220	69.21	1.361			
**Pd** **_1_** **Cu** **_(d)_** **-NHGS**	111	40.75	2.220	3.820	2.04: 1	71.91
	220	69.52	1.356			

**Table S2:** Comparison of current density and ECSA for EOR using different Pd-based electrocatalysts in alkaline medium.

Catalysts	Scan rate (mV/s)	ECSA (m ^2^/g)	Current density (mA/mg)	Reference	Year
Pd _68_Ni _32_/rGO	50	142	1820	[ 9]	2018
Pd _3_NiP/N-rGO	50	83	2223	[ 11]	2020
PdAu NW	50	96	2253	[ 18]	2021
Pd@Cd _3_-Ag _1_	50	78	2928	[ 19]	2021
FePd-Fe _2_O _3(3:5)_/MWNTs	50	120	1191	[ 23]	2015
PdCu-3	50	25	1230	[ 24]	2020
PdCu _(F)_-RGO	50	152	2416	[ 45]	2017
Pd _4_Cu _(d)_-NHGS	50	59	2683	This paper	--
10% Pd/C	50	24	1035	--	--
