# Supplementary figures and images for: Fabrication of dendritic PdCu alloy supported on 3D N-doped hollow graphene for efficient ethanol electrooxidation
Source: Turk J Chem. 2022 Nov 29;47(1):207–17. doi: 10.55730/1300-0527.3530 (PMC10504011; doi:10.55730/1300-0527.3530)

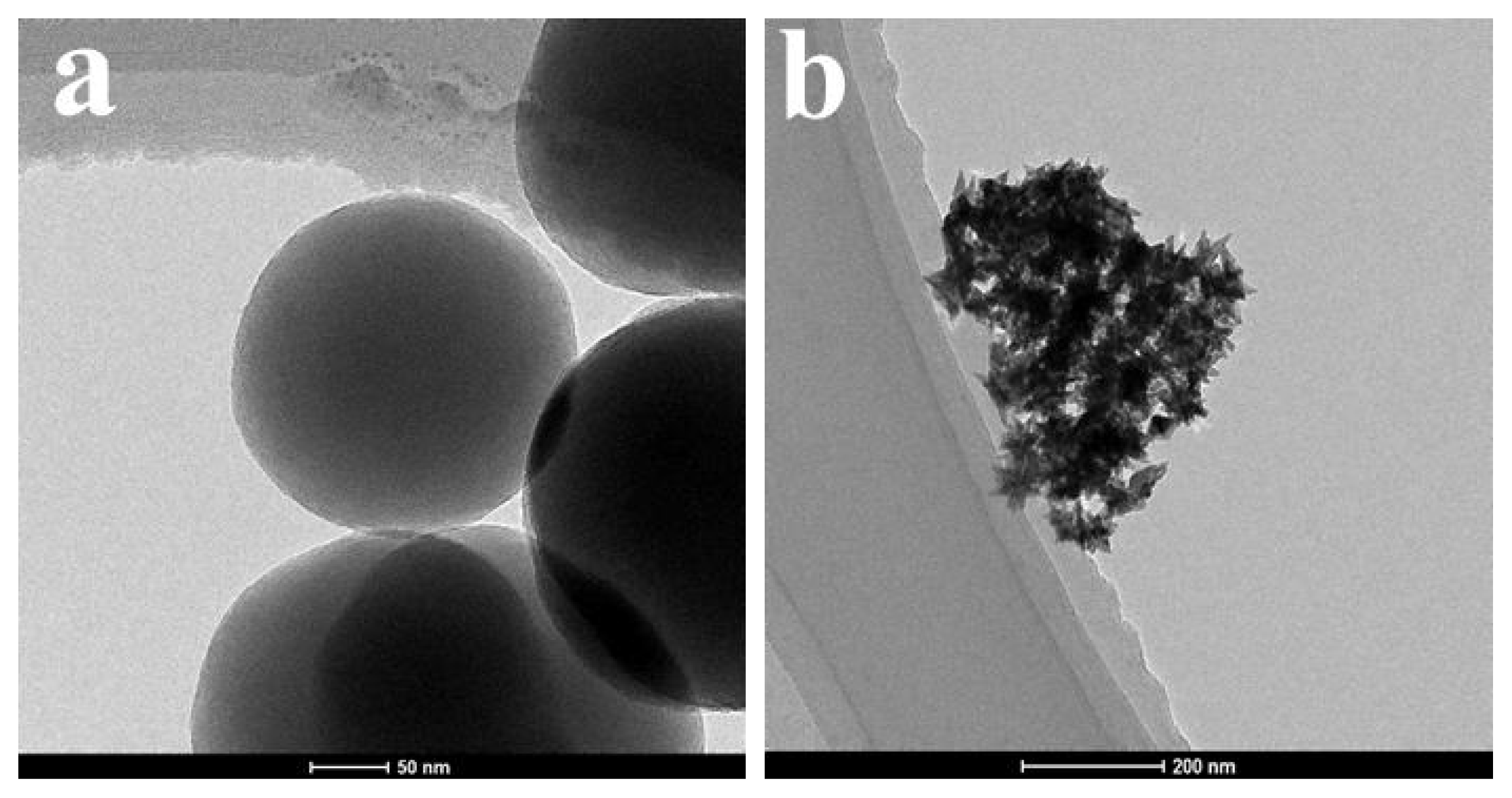

Supplement: Figure S1 — TEM images of SiO2 nanospheres (a) and Pd4Cu(d) alloys (b). [file turkjchem-47-1-207s1.tif]

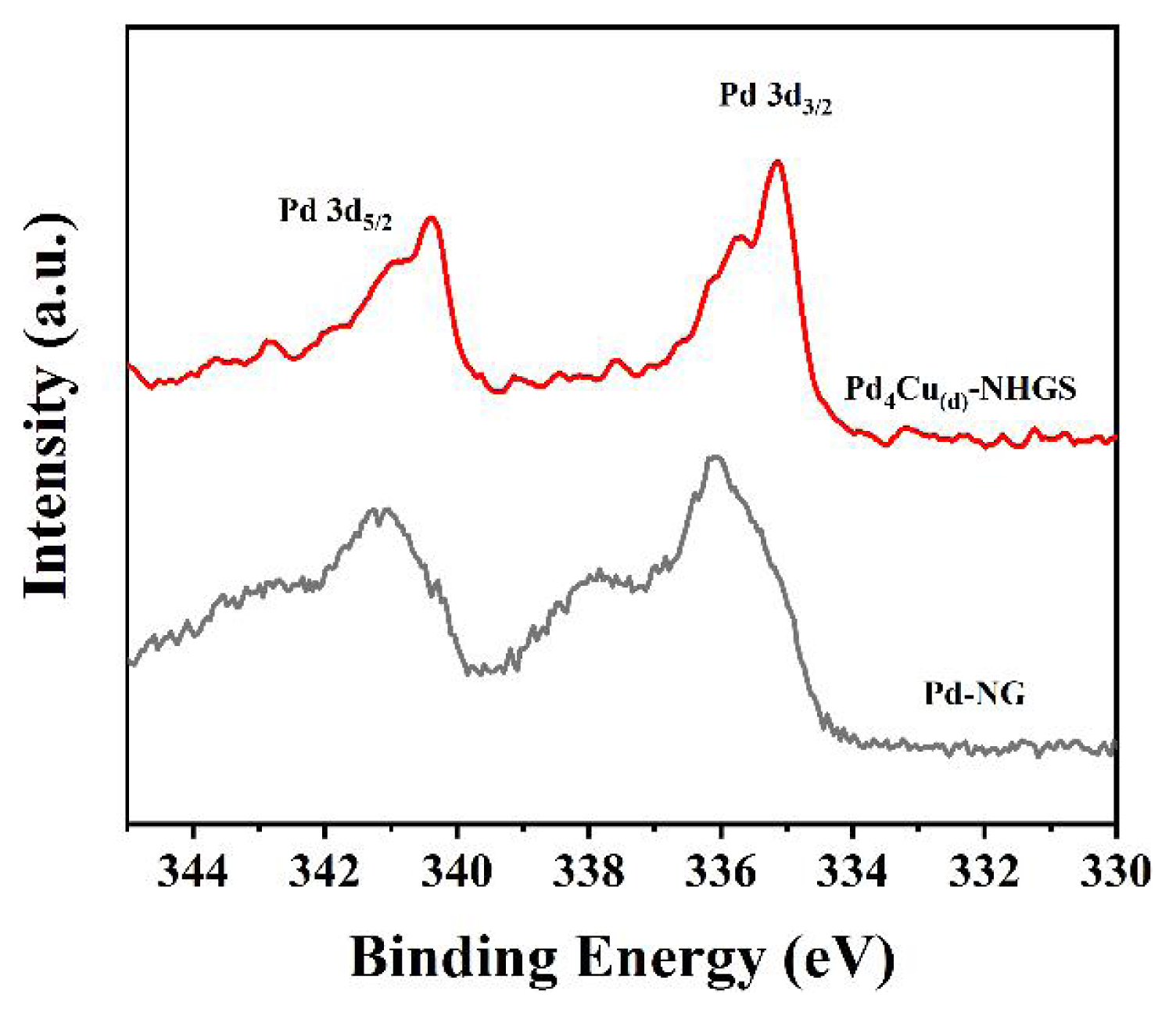

Supplement: Figure S2 — XPS spectra of Pd4Cu(d)-NHGS and Pd-NG. [file turkjchem-47-1-207s2.tif]
